# Image quality and compression force: the forgotten link in optimisation of digital mammography?

**DOI:** 10.1186/bcr2962

**Published:** 2011-11-04

**Authors:** D O'Leary, T Grant, L Rainford

**Affiliations:** 1 UCD, School of Medicine and Medical Science, Dublin, Ireland

## Introduction

Numerous publications suggest the compression force applied in mammography must be reduced to encourage initial/continued attendance by women. These studies (many with limited patient populations) have not used sufficient statistical correlation of compression force data to image quality to reinforce these contentions. The growth of digital mammography has enabled a comparison of compression force and image quality in analogue and digital images.

## Methods

A quantitative and qualitative study of mammography units within Ireland collected comprehensive image quality, compression (force and depth) and radiation dose data from 4,790 patient images. The data were analysed using univariate analysis of variance and SPSS statistics including ANOVA to re-examine the connections between all parameters for optimisation of mammographic imaging.

## Results

The amount of compression force consistently showed significant effects on the image quality; perfect and good images consistently required significantly more compression force than moderate and inadequate images. This was especially apparent in digital images. The mean compression force (Newtons) required to produce a perfect image was: 121.34 N for digital craniocaudal; 134.23 N for digital mediolateral oblique; 112.23 N for analogue craniocaudal; and 129.66 N for analogue mediolateral oblique. Only 2% of patients expressed dissatisfaction with the higher compression forces that were applied.

## Conclusion

Compression forces are too low, affecting image quality; greater compression force by 11 to 15 N is needed to achieve a perfect image especially in full-field digital mammography. Greater training of radiographers performing mammography is required to standardise the undertaking of the mammographic projections with regard to achievable compression depth and the application of compression force delivered to the breasts of Irish women attending the symptomatic breast services.

